# Oral high dose ascorbic acid treatment for one year in young CMT1A patients: a randomised, double-blind, placebo-controlled phase II trial

**DOI:** 10.1186/1741-7015-7-70

**Published:** 2009-11-12

**Authors:** Camiel Verhamme, Rob J de Haan, Marinus Vermeulen, Frank Baas, Marianne de Visser, Ivo N van Schaik

**Affiliations:** 1 Department of Neurology and Clinical Neurophysiology, Academic Medical Centre, University of Amsterdam, PO Box 22660, 1100 DD, Amsterdam, The Netherlands; 2 Department of Clinical Epidemiology and Biostatistics, Academic Medical Centre, University of Amsterdam, PO Box 22660, 1100 DD, Amsterdam, The Netherlands

## Abstract

**Background:**

High dose oral ascorbic acid substantially improved myelination and locomotor function in a Charcot-Marie-Tooth type 1A mouse model. A phase II study was warranted to investigate whether high dose ascorbic acid also has such a substantial effect on myelination in Charcot-Marie-Tooth type 1A patients and whether this treatment is safe.

**Methods:**

Patients below age 25 years were randomly assigned to receive placebo or ascorbic acid (one gram twice daily) in a double-blind fashion during one year. The primary outcome measure was the change over time in motor nerve conduction velocity of the median nerve. Secondary outcome measures included changes in minimal F response latencies, compound muscle action potential amplitude, muscle strength, sensory function, Charcot-Marie-Tooth neuropathy score, and disability.

**Results:**

There were no significant differences between the six placebo-treated (median age 16 years, range 13 to 24) and the five ascorbic acid-treated (19, 14 to 24) patients in change in motor nerve conduction velocity of the median nerve (mean difference ascorbic acid as opposed to placebo treatment of 1.3 m/s, confidence interval -0.3 to 3.0 m/s, *P *= 0.11) or in change of any of the secondary outcome measures over time. One patient in the ascorbic acid group developed a skin rash, which led to discontinuation of the study medication.

**Conclusion:**

Oral high dose ascorbic acid for one year did not improve myelination of the median nerve in young Charcot-Marie-Tooth type 1A patients. Treatment was relatively safe.

**Trial registration:**

Current Controlled Trials ISRCTN56968278, ClinicalTrials.gov NCT00271635.

## Background

Charcot-Marie-Tooth type 1A (CMT1A), also known as Hereditary Motor and Sensory Neuropathy type Ia (HMSN Ia), is the most prevalent hereditary peripheral neuropathy (approximately 1:5,000 people) [1]. CMT1A is an autosomal dominant disease, most often caused by a 1.5 Mb duplication on chromosome 17, giving rise to three copies of the peripheral myelin protein 22 (*PMP22*)-gene. Histologically, there are signs of dysmyelination, of active de- and remyelination especially during the first years of life, and of axonal loss [2,3]. The conduction velocities of the peripheral nerves are slow very early in life and after the age of approximately five years longitudinal data show that the conduction velocities remain fairly stable, consistent with a fairly constant myelination status [4-7]. Compound muscle action potential (CMAP) amplitudes as recorded from distal leg muscles are found to decrease during the first two decades of life, indicating increasing secondary axonal dysfunction [8]. This increasing axonal dysfunction is responsible for the development of symptoms and signs of predominantly distal muscle weakness, atrophy, and sensory abnormalities, in the legs more than the arms [8,9]. One of the factors determining the severity of secondary axonal dysfunction is the degree of initial abnormal myelination [9]. Until now, therapy is symptomatic and aims at maintaining functional abilities and learning compensation mechanisms. Therapy directed at restoration of myelination is expected to be most beneficial in young patients, as more secondary axonal dysfunction may be prevented than in older patients [7].

An *in vitro *study showed that high doses of ascorbic acid may repress *PMP22 *gene expression by acting on intracellular cyclic adenosine monophosphate levels and adenylate cyclase activity [10]. This may explain why in a mouse model for CMT1A, in which the animals over-express human *PMP22*, ascorbic acid induced remyelination and improved locomotor functioning [11]. Mice aged two to four months treated with orally administered high doses of ascorbic acid once a week during three months showed increased percentages of myelinated fibres (from 25 to 70%) and increased myelin sheath thickness in the sciatic nerves with fairly normal g ratios [11]. Electrophysiological data have not been published in this study, but the substantial improvements of myelination parameters in the histological analysis suggested that the same may hold true for nerve conduction. Nerve conduction velocities are a non-invasive surrogate marker for myelination which are relatively easily obtainable in longitudinal studies in CMT1A patients [4-7]. For this reason, we initiated a proof of principle study to investigate whether ascorbic acid also has a substantial effect on myelination in CMT1A patients. This may in turn also have a positive effect on axonal function and in that respect on clinical impairments and disability. Therefore, we performed a single-centre, randomised, double-blinded, phase II study comparing a high dose ascorbic acid with a placebo during one year to assess the effects of ascorbic acid on myelination and its safety in young CMT1A patients.

## Methods

CMT1A patients were recruited from the outpatient department of neurology at the Academic Medical Centre (AMC), University of Amsterdam and via the Dutch Neuromuscular Patient Association (Vereniging Spierziekten Nederland). They were assessed for eligibility between December 2005 and June 2006. Inclusion criteria were: DNA-proven diagnosis of CMT1A; age range 12 to 25 years; clinically affected status defined as muscle weakness of at least the foot dorsiflexors. Exclusion criteria were: other known disease or medication that may cause a neuropathy, chronic alcohol abuse, regular use of ascorbic acid before the study, clinical or ultrasound signs of nephrolithiasis, reduced glomerular filtration rate, iron overload, no regular dental control at the dentist, pregnancy or active pregnancy wish for women, psychiatric co-morbidity which may influence compliance, reluctance to undergo nerve conduction studies, a low CMAP amplitude of the abductor pollicis brevis muscle (negative peak < 0.5 mV) hindering proper determination of the motor nerve conduction velocity (MNCV) of the median nerve.

The local medical ethics committee approved the study protocol. All patients, and where appropriate the parents, gave written informed consent to participate in the study. This study has been registered in ClinicalTrials.gov, number NCT00271635, and in Current Controlled Trials, number ISRCTN56968278.

### Procedure

Eligible patients were randomly allocated to oral ascorbic acid or placebo. The pharmacist, who did not have any further role in the study, generated a computerized randomization sequence. Participants and investigators were unaware of the treatment assignment. The tolerable upper intake level of ascorbic acid for adults is 2 g/day, for children 14 to 18 years 1.8 g/day, and for children 9 to 13 years 1.2 g/day. A higher intake is acceptable with clinical monitoring [12]. Treatment was administered in a double-blind fashion for 12 months and was either four capsules containing 250 mg ascorbic acid twice daily (daily dose: 2 g), or four capsules containing placebo with identical size, colour, shape, and taste twice daily. Patients were assessed for all outcome measures at baseline and at 6 and 12 months follow-up by the same investigator (C.V.). Given the explorative nature of the phase II study, we used a primary outcome measure at the impairment level to test the efficacy of ascorbic acid; the mean change over time of the MNCV of the median nerve at 12 months follow-up, which we consider the best non-invasive surrogate marker for changes in myelination [9,13,14]. At the non-dominant side of the body large surface recording electrodes (2.0 × 2.6 cm) were attached over the surface of the abductor pollicis brevis muscle at standardised sites defined by anatomical landmarks. Temperature was maintained between 32 and 34°C as measured at distal recording sites. The median nerve was stimulated at the wrist and elbow, and the MNCV was calculated. The secondary outcomes included changes over time at 12 months of other measures at the impairment and disability level. The mean change over time of the minimal F response latency of the median nerve was an additional non-invasive surrogate marker for changes in myelination. The mean change over time of the CMAP amplitude (negative peak) recorded from the abductor pollicis brevis muscle was a non-invasive surrogate marker for changes in axonal function [9,14]. The mean change over time of strength of the three point grip and the foot dorsiflexors as assessed with hand-held dynamometry were indicators for changes in arm and leg strength, respectively [15,16]. The median change over time of the Inflammatory neuropathy cause and treatment sensory sum score (ISS) assessed changes in sensory function [17]. It comprises vibration and pinprick sense plus a two-point discrimination value and ranges from 0 (normal sensation) to 20 (maximum sensory deficit). The median change over time of the Charcot-Marie-Tooth neuropathy score (CMTNS) assessed changes in an overall composite impairment score [18]. It comprises items from history, clinical and electrophysiological investigations and ranges from 0 (no deficit) to 36 (maximal deficit). The mean change over time in the AMC linear Disability Score (ALDS) was an indicator for changes in disability [19]. It ranges from 0 (dead) to 100 (fully able). The median change over time of the overall disability sum score (ODSS) was another indicator for changes in disability [20]. It comprises arm and leg items and ranges from 0 (no disability) to 12 (maximum disability). Indicators for changes in functional performance were the mean change over time of the time needed for the nine hole peg test [16,21], and of the time needed for the 50 m walking test, which we decided would be more sensitive to change than the 10 m walking test [16]. Motor unit number estimation of the thenar muscles was determined in the context of another study [22], and will be reported separately.

Adverse events and laboratory values were monitored every three months or more often if necessary, and were reviewed by an independent Data Safety Monitoring Board. A repeat ultrasound of the kidneys was done at 12 months. Capsule counts were performed every three months to monitor treatment adherence. At the end of the study the success of blinding was evaluated by asking the participant and the investigator separately which trial medication they thought the participant had used (placebo, ascorbic acid or 'I really don't know').

### Statistical analysis

We deduced from the substantial improvement in histological myelination parameters of the animal experimental study that at least a mean improvement of the MNCV of the median nerve of 10 m/s should be detectable in the ascorbic acid-treated group as opposed to the placebo-treated group. Such an improvement was considered potentially clinically meaningful. In a previous study on 46 CMT1A patients, the mean MNCV of the median nerve was found to be 23 m/s with a standard deviation (SD) of 5.3 [9]. As there were no SDs for possible changes in MNCV, we conservatively used SDs of 5.3 to calculate group sizes. Six patients per group were required to detect a difference of 10 m/s with 80% power and a two-sided alpha level of 0.05 using the unpaired *t *test. Randomised patients with at least one follow-up measure of the primary outcome were analyzed according to the intention-to-treat principle. In case of incomplete data, we used the last observation carried forward approach. The mean or median change scores from baseline to final follow-up of the patients receiving ascorbic acid and those receiving placebo were presented together with the differences in these change scores between the treatment groups using unpaired *t *test or Mann-Whitney *U *test, when appropriate. Statistical uncertainty was quantified via corresponding 95% confidence intervals for the differences in change scores between the treatment groups. Additionally, we analysed the MNCV of the median nerve at follow-up, given the MNCV of the median nerve at baseline, using analysis of covariance to evaluate the effect of being treated either with ascorbic acid or placebo [23]. For the analysis of covariance we presented the averaged difference together with the 95% confidence interval. All analyses were done with SPSS 15.0 for Windows (SPSS Inc. Chicago, Illinois, USA).

## Results

**Figure 1:**
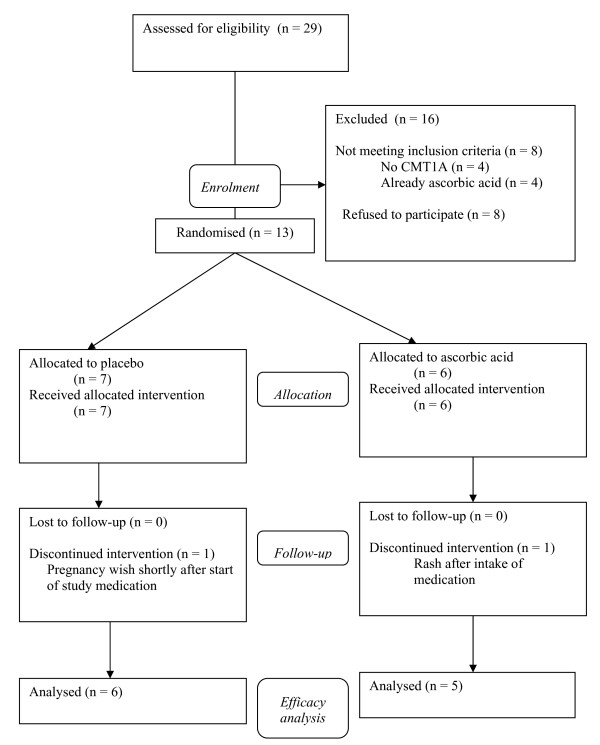
**Flow diagram of the trial**.

Sixteen of the 29 patients initially screened for the study did not meet the inclusion criteria (Figure 1). Eight patients refused to participate for various reasons. Four patients, who responded via the patients' association, had another form of CMT. Four patients already used supplemental ascorbic acid. From the randomised 13 patients one female participant in the placebo group aged 18 years with a baseline MNCV of the median nerve of 21 m/s was excluded during the study because of a pregnancy wish before the six months' assessment. One male participant in the ascorbic acid group aged 21 years with a baseline MNCV of the median nerve of 16 m/s developed a skin rash after the first days of study medication use which disappeared after stopping the study medication.

Baseline characteristics of the 11 patients in which at least one primary outcome measure during follow-up was obtained are described in Table 1. Both groups were fairly similar with regard to baseline characteristics. In each group, one participant had a follow-up until six months: in the placebo group one participant discontinued medication due to gastrointestinal complaints and in the ascorbic acid group one participant failed to attend the outpatient clinic afterwards. All the other participants had a follow-up until 12 months.

**Table 1:** Baseline characteristics of study participants described for primary and secondary outcome measures.

	Placebo (n = 6)	Ascorbic acid (n = 5)
Male/Female (numbers)	2/4	3/2
Age (years)^b^	16 (13-24)	19 (14-24)
		
Median nerve MNCV (m/s)^a^	17.9 (6.0)	17.2 (1.7)
Median nerve minimal F response latency (ms)^a^	83.8 (28.0)*	77.1 (10.6)
CMAP amplitude abductor pollicis brevis muscle (mV)^a^	3.9 (1.1)	4.0 (1.2)
Three point grip (N)^a^	50.9 (14.4)	73.8 (24.7)
Foot dorsiflexors (N)^a^	79.5 (14.0)	91.1 (26.9)
ISS^b^	2 (0-8)	3 (1-6)
CMTNS^b^	10 (7-20)	13 (7-17)
ODSS^b^	3 (2-4)	2 (2-4)
ALDS^a^	86.8 (6.5)	89.0 (0.5)
Nine hole peg test (s)^a^	26.3 (6.6)	23.5 (1.8)
50 m walking (s)^a^	31.9 (2.0)	29.2 (3.2)

^a ^Mean (standard deviation) ^b^Median (range) * n = 5

Abbreviations: ALDS = Academic Medical Centre linear disability score; CMAP = Compound muscle action potential; CMTNS = Charcot-Marie-Tooth neuropathy score; ISS = inflammatory neuropathy cause and treatment sensory sum score; MNCV = motor nerve conduction velocity; ODSS = overall disability sum score

**Figure 2:**
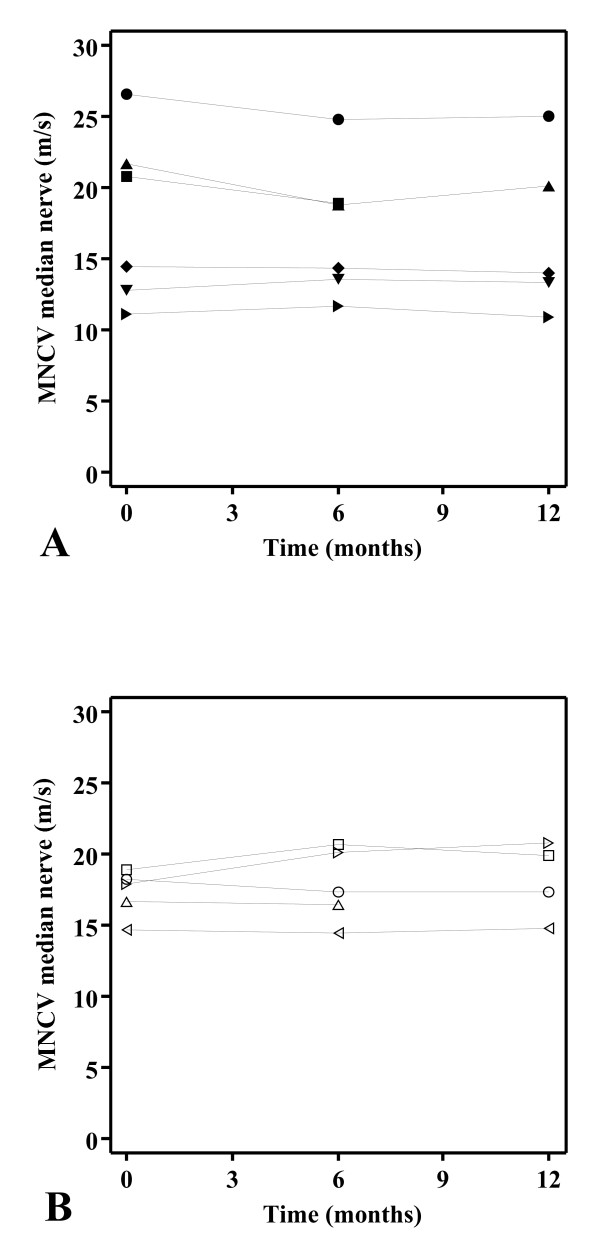
**Motor nerve conduction velocities of the median nerve over time**. Motor nerve conduction velocity (MNCV) of the median nerve (m/s) over time (months) in the placebo (A) and ascorbic acid (B) treated participants. Each connected symbol corresponds with an individual participant.

The MNCVs of the median nerve over time of individual participants in the placebo and ascorbic acid treated groups are shown in figure 2A and 2B, respectively. Table 2 summarizes the primary and secondary outcome measures. The mean change in MNCV of the median nerve over time was not different between the treatment groups (*P *= 0.11). Additionally, analysis of covariance showed a non-significant averaged difference of 1.3 m/s (confidence interval -0.4 to 2.9, *P *= 0.12) in favour of the ascorbic acid as opposed to the placebo treated group, given the MNCVs of the median nerve at baseline. None of the secondary outcome measures showed significant score changes.

**Table 2:** Primary and secondary treatment outcomes according to assigned treatment.

	Placebo (n = 6)	Ascorbic acid (n = 5)		
				
	Change	Change	Difference	*P *value
Median nerve MNCV (m/s)^a^	-0.8 (0.9)	0.5 (1.5)	1.3 (-0.3-3.0)	0.11
Median nerve minimal F response latency (ms)^a^	3.5 (8.0)*	-5.6 (4.4)	-9.1 (-18.5-0.3)	0.06
CMAP amplitude abductor pollicis brevis muscle (mV)^a^	-0.02 (0.9)	-0.2 (0.8)	-0.1 (-1.3 -1.1)	0.79
Three point grip (N)^a^	-3.0 (9.2)	3.4 (9.0)	6.4 (-6.1-18.9)	0.28
Foot dorsiflexors (N)^a^	-5.1 (17.6)	0.6 (15.6)	5.6 (-17.3-28.6)	0.59
ISS^b^	0 (-4-1)	-2 (-2-1)	-2 (-2-2)	0.30
CMTNS^b^	1 (-5-4)	-2 (-3-1)	-3 (-6-3)	0.14
ODSS^b^	0 (0-1)	0 (-2-0)	0 (-2-0)	0.18
ALDS^a^	1.0 (3.7)	0.3 (0.4)	-0.6 (-4.5-3.2)	0.30
Nine hole peg test (s)^a^	-3.5 (2.8)	-2.0 (1.8)	1.5 (-1.8-4.8)	0.32
50 m walking (s)^a^	-0.6 (1.9)	-0.9 (1.8)	-0.3 (-2.9-2.2)	0.77

Change = ^a ^mean (standard deviation) or ^b^median (range) change scores from baseline to final follow-up. Difference = difference in ^a ^mean or ^b ^median change scores (95% confidence interval) of the patients receiving ascorbic acid and those receiving placebo. *P*-value = *P*-value for ^a ^the unpaired *t *test (equal variances assumed) or ^b^the Mann-Whitney *U *test. * n = 5

Abbreviations: ALDS = Academic Medical Centre linear disability score; CMAP = compound muscle action potential; CMTNS = Charcot-Marie-Tooth neuropathy score; ISS = inflammatory neuropathy cause and treatment sensory sum score; MNCV = motor nerve conduction velocity; ODSS = overall disability sum score

Adverse events were registered for all randomised participants (n = 13) who were allocated trial medication. In the placebo group three out of the seven included participants had gastrointestinal complaints during the study, while in the ascorbic acid group one of the six participants had an adverse event. In the placebo group one female participant had severe complaints of cramps and diarrhoea to such an extent that she discontinued the study medication after just six months, one participant had feelings of nausea during the nine months and 12 months visits, and one participant had feelings of nausea and cramps at three months. The latter individual was excluded from the study because of a pregnancy wish just after three months (Figure 1). In the ascorbic acid group one adult participant developed a skin rash after the first days of treatment (see also Figure 1). This was interpreted as an allergic reaction; the rash disappeared after stopping the study medication. None of the 13 participants complained about renal colic or reported tooth problems. There were no relevant changes in laboratory values. None of the nine participants who attended at 12 months had signs of kidney stones or renal disorders on a repeat ultrasound of the kidneys. Of the participants who left the study before 12 months no repeat ultrasound was made.

Counts of returned capsules showed that the six placebo treated participants and the five ascorbic acid treated participants both used 88% of the capsules on average during the study period. In the ascorbic acid group three out of five participants did not know what treatment they were using, one correctly and one incorrectly identified group assignment. In the placebo group four out of six participants did not know their treatment allocation and two correctly identified group assignment. The investigator did not know treatment allocation of any participant. Forced guessing corresponded with correct group allocation in three out of five in the ascorbic acid group and four out of six in the placebo group. None of the participants reported the intake of supplemental ascorbic acid during the study period.

## Discussion

In this phase II study high dose ascorbic acid given during one year to young CMT1A patients did not lead to a positive predefined effect on myelination, assessed as a difference of the MNCV of the median nerve of 10 m/s between the ascorbic acid- and placebo-treated patients. We found a non-significant difference in mean MNCV change scores between ascorbic acid as opposed to placebo treatment of 1.3 m/s with an upper limit of the confidence interval of 3 m/s. Neither could we demonstrate significant effects of ascorbic acid for any of the secondary outcome measures, including minimal F response latency of the median nerve, CMAP amplitude, muscle strength, sensory function and disability. The treatment consisting of 2 g per day during one year was relatively safe, although there was one serious adverse event: one patient in the ascorbic acid group developed a rash, which led to discontinuation of the study medication. A delayed type hypersensitivity to ascorbic acid has been seldom reported [24].

The rationale for our study was derived from the results of a study, showing a robust effect of high doses ascorbic acid on myelination in a *PMP22 *over-expressing mouse model [11]. To our knowledge these results have not been reproduced in the same or other animal models. A 12-month randomised, double-blind, placebo controlled trial in 81 CMT1A children aged 2 to 16 years with comparable high-dose oral ascorbic acid (30 mg/kg/day) did not show an improvement on motor nerve conduction velocity, CMAP amplitudes or functional abilities but treatment was safe and well tolerated [25]. A two-year non-randomised open-label pilot study compared a group of 12 adult patients starting with 5 g of ascorbic acid daily with a group of 10 age-matched untreated patients [26]. Five out of 12 treated patients (42%) tolerated 5 g daily for the entire study period; two (17%) were able to finish the study period after lowering the dose to 2.5 g daily; five (42%) discontinued treatment, mainly due to gastrointestinal side effects. Nerve conduction velocities, CMAP amplitudes and the CMTNS did not improve over time when either the whole group of 12 patients initially receiving ascorbic acid, or the five patients who received 5 g daily during the entire study period, were compared with the control group [26]. This study showed that adverse events limit the tolerable doses in CMT1A patients and did not provide evidence for the effectiveness of doses as high as 5 g daily.

The discrepancy in effectiveness of ascorbic acid between the mouse model and humans may have several explanations. *PMP22 *gene over-expression may be higher in the mouse model, as this model has seven extra copies of the human *PMP22 *gene, while human patients only have one extra copy. Thus, myelination is more severely affected in the mouse model, as indicated by a considerably lower mean MNCV in the mouse model as compared to that in CMT1A patients [9,27]. Whether this difference in myelination of the peripheral nerves at outset evokes a different response to high dose ascorbic acid is unknown. The effectiveness of *PMP22 *gene repression in humans may be too small to notice a clear effect on myelination. Another explanation may be the fact that ascorbic acid metabolism in humans is distinct from that in mice. Humans derive ascorbic acid entirely from dietary sources, while mice are able to synthesize ascorbic acid [28]. There may also be a difference between mice and humans with regard to the uptake of ascorbic acid or its metabolic intermediates at the tissue and cellular level, leading to different effects.

Some strengths of this study should be addressed. To answer the question whether the substantial positive effects of high dose ascorbic acid on myelination in a CMT1A mouse model may be reproducible in humans a non-invasive surrogate marker for myelination (MNCV of the median nerve) was chosen as primary efficacy outcome measure because this measure reflects the intended biological effect (induction of remyelination) as close as possible. MNCVs are validated measures [14] and commonly used as a non-invasive surrogate marker for myelination in CMT1A studies [4-7]. We assumed that a follow-up of one year should be long enough to detect at least any indication of remyelination. Theoretically, this remyelination should secondarily lead to a decrease in axonal dysfunction. This introduces more uncertain factors like the capacity of remyelination to prevent axonal dysfunction and even to restore axonal function over time in CMT1A patients. This is why parameters like the composite CMTNS were included as secondary outcome measures. It is of note that the CMTNS does not include any measure of myelination, and thus was considered not to be suitable as the primary outcome measure in our study. Finally, we included young patients who may benefit most from treatment, as more axonal dysfunction may be prevented than in older patients.

Some limitations of this study should also be addressed. In retrospect, the conversion from the improvement of myelination parameters in the histological analysis in the mouse study [11] to the intended improvement in humans, 10 m/s for the MNCV of the median nerve, may have been too optimistic. In CMT1A there are no data available about the minimal increase of MNCVs giving rise to a clinically meaningful secondary effect on axonal function and more importantly on disability. However, we hypothesized that a 10 m/s increase was potentially clinically relevant and we performed a power calculation based on this, leading to small numbers of patients in each group. Since our goal was to find out whether high dose ascorbic acid had the same substantial effect on myelination as had been found in the animal study, we decided to carry out a small proof of principle study first. The non-significant difference in mean MNCV change scores between ascorbic acid and placebo treated patients we found is in accordance with the non-significant difference in a randomised controlled trial in a much larger group of patients [25] and is in our opinion too little to have enough potential clinical relevance to initiate studies with larger numbers of patients, although we are aware that these are under way [29]. Moreover, the difference we found may well be due to normal measurement variation inherent to conduction studies as suggested by the fact that the major part of this modest difference was due to a decrease in MNCV over time in the placebo group and not to an increase in MNCV over time in the ascorbic acid group.

## Conclusion

This randomised double-blind, placebo-controlled, phase II trial with high dose ascorbic acid (2 g per day) for one year in children and young adults with CMT1A was initiated as a proof of principle study but did not show an improvement of myelination in the median nerve. In our hands treatment with 2 g per day during one year was relatively safe. Thus, evidence is accumulating that ascorbic acid does not have the same robust positive effect on myelination and functional abilities as was shown in the *PMP22 *over-expressing mouse model [11] in neither child nor (young) adult CMT1A patients [25,26].

## Abbreviations

ALDS: Academic Medical Centre linear disability score; AMC: Academic Medical Centre; CMAP: compound muscle action potential; CMT: Charcot-Marie-Tooth; CMTNS: Charcot-Marie-Tooth neuropathy score; CMT1A: Charcot-Marie-Tooth type 1A; HMSN Ia: Hereditary Motor and Sensory Neuropathy type Ia; ISS: inflammatory neuropathy cause and treatment sensory sum score; MNCV: motor nerve conduction velocity; ODSS: overall disability sum score; PMP22: peripheral myelin protein 22; SD: standard deviation.

## Competing interests

The authors declare that they have no competing interests.

## Authors' contributions

CV participated in the design of the study, carried out the study, performed the statistical analysis and drafted the manuscript. RdH helped with the statistical analysis and helped to draft the manuscript. MV, FB and MdV participated in its design and helped to draft the manuscript. IvS participated in its design and coordination, helped with the statistical analysis and helped to draft the manuscript. All authors read and approved the final manuscript.

## Pre-publication history

The pre-publication history for this paper can be accessed here:

http://www.biomedcentral.com/1741-7015/7/70/prepub

## References

[B1] Nelis E Van Broeckhoven C De Jonghe P Lofgren A Vandenberghe A Latour P Le Guern E Brice A Mostacciuolo ML Schiavon F Palau F Bort S Upadhyaya M Rocchi M Archidiacono N Mandich P Bellone E Silander K Savontaus ML Navon R Goldberg-Stern H Estivill X Volpini V Friedl W Gal A Estimation of the mutation frequencies in Charcot-Marie-Tooth disease type 1 and hereditary neuropathy with liability to pressure palsies: a European collaborative study Eur J Hum Genet 1996 4 25 33 8800924 10.1159/000472166

[B2] Gabreëls-Festen AA Joosten EM Gabreëls FJ Jennekens FG Janssen-van Kempen TW Early morphological features in dominantly inherited demyelinating motor and sensory neuropathy (HMSN type I) J Neurol Sci 1992 107 145 154 10.1016/0022-510X(92)90282-P 1564512

[B3] Robaglia-Schlupp A Pizant J Norreel JC Passage E Saberan-Djoneidi D Ansaldi JL Vinay L Figarella-Branger D Levy N Clarac F Cau P Pellissier JF Fontés M PMP22 overexpression causes dysmyelination in mice Brain 2002 125 2213 2221 10.1093/brain/awf230 12244079

[B4] Garcia A Combarros O Calleja J Berciano J Charcot-Marie-Tooth disease type 1A with 17p duplication in infancy and early childhood: a longitudinal clinical and electrophysiologic study Neurology 1998 50 1061 1067 9566395 10.1212/wnl.50.4.1061

[B5] Killian JM Tiwari PS Jacobson S Jackson RD Lupski JR Longitudinal studies of the duplication form of Charcot-Marie-Tooth polyneuropathy Muscle Nerve 1996 19 74 78 10.1002/(SICI)1097-4598(199601)19:1<74::AID-MUS10>3.0.CO;2-3 8538673

[B6] Shy ME Chen L Swan ER Taube R Krajewski KM Herrmann D Lewis RA McDermott MP Neuropathy progression in Charcot-Marie-Tooth disease type 1A Neurology 2008 70 378 383 10.1212/01.wnl.0000297553.36441.ce 18227419

[B7] Verhamme C van Schaik IN Koelman JH De Haan RJ de Visser M The natural history of Charcot-Marie-Tooth type 1A in adults: a 5-year follow-up study Brain 2009 in press 19843647 10.1093/brain/awp251

[B8] Berciano J Garcia A Calleja J Combarros O Clinico-electrophysiological correlation of extensor digitorum brevis muscle atrophy in children with Charcot-Marie-Tooth disease 1A duplication Neuromuscul Disord 2000 10 419 424 10.1016/S0960-8966(99)00114-5 10899448

[B9] Verhamme C van Schaik IN Koelman JH De Haan RJ Vermeulen M De Visser M Clinical disease severity and axonal dysfunction in hereditary motor and sensory neuropathy Ia J Neurol 2004 251 1491 1497 10.1007/s00415-004-0578-x 15645349

[B10] Kaya F Belin S Bourgeois P Micaleff J Blin O Fontes M Ascorbic acid inhibits *PMP22 *expression by reducing cAMP levels Neuromuscul Disord 2007 17 248 253 10.1016/j.nmd.2006.12.008 17303424

[B11] Passage E Norreel JC Noack-Fraissignes P Sanguedolce V Pizant J Thirion X Robaglia-Schlupp A Pellissier JF Fontes M Ascorbic acid treatment corrects the phenotype of a mouse model of Charcot-Marie-Tooth disease Nat Med 2004 10 396 401 10.1038/nm1023 15034573

[B12] Food and Nutrition Board Institute of Medicine National Academy of Sciences Dietary reference intakes for vitamin C, Vitamin E, Selenium, and Carotenoids 2000 Washington DC: National Academy Press http://www.nap.edu/catalog.php?record_id=9810 25077263

[B13] van Dijk JG Kamp WIM van der van Hilten BJ van Someren P Influence of recording site on CMAP amplitude on its variation over a length of nerve Muscle Nerve 1994 17 1286 1292 10.1002/mus.880171107 7935551

[B14] Tjon-A-Tsien AM Lemkes HH Kamp-Huyts AJ van der van Dijk JG Large electrodes improve nerve conduction repeatability in controls as well as in patients with diabetic neuropathy Muscle Nerve 1996 19 689 695 10.1002/(SICI)1097-4598(199606)19:6<689::AID-MUS1>3.0.CO;2-6 8609917

[B15] Ploeg RJ van der Fidler V Oosterhuis HJ Hand-held myometry: reference values J Neurol Neurosurg Psychiatry 1991 54 244 247 2030353 10.1136/jnnp.54.3.244 PMC1014394

[B16] Solari A Laura M Salsano E Radice D Pareyson D Reliability of clinical outcome measures in Charcot-Marie-Tooth disease Neuromuscul Disord 2008 18 19 26 10.1016/j.nmd.2007.09.006 17964785

[B17] Merkies IS Schmitz PI Meché FG van der van Doorn PA Psychometric evaluation of a new sensory scale in immune-mediated polyneuropathies. Inflammatory Neuropathy Cause and Treatment (INCAT) Group Neurology 2000 54 943 949 10690990 10.1212/wnl.54.4.943

[B18] Shy ME Blake J Krajewski K Fuerst DR Laura M Hahn AF Li J Lewis RA Reilly M Reliability and validity of the CMT neuropathy score as a measure of disability Neurology 2005 64 1209 1214 15824348 10.1212/01.WNL.0000156517.00615.A3

[B19] Weisscher N Post B De Haan RJ Glas CAW Speelman JD Vermeulen M The AMC Linear Disability Score in patients with newly diagnosed Parkinson disease Neurology 2007 69 2155 2161 10.1212/01.wnl.0000295666.30948.9d 18056579

[B20] Merkies IS Schmitz PI Meche FG van der Samijn JP van Doorn PA Clinimetric evaluation of a new overall disability scale in immune mediated polyneuropathies J Neurol Neurosurg Psychiatry 2002 72 596 601 11971045 10.1136/jnnp.72.5.596 PMC1737884

[B21] Mathiowetz V Weber K Kashman N Volland G Adult norms for the nine hole peg test of finger dexterity Occupational Therapy J Research 1985 5 24 38 http://psycnet.apa.org/?fa=main.doiLanding&uid=1986-05316-001 10.5014/ajot.39.6.386 3160243

[B22] van Dijk JP Blok JH Lapatki BG van Schaik IN Zwarts MJ Stegeman DF Motor unit number estimation using high-density surface electromyography Clinical Neurophysiology 2008 119 33 42 10.1016/j.clinph.2007.09.133 18037342

[B23] Vickers AJ Altman DG Statistics Notes: Analysing controlled trials with baseline and follow up measurements BMJ 2001 323 1123 1124 11701584 10.1136/bmj.323.7321.1123 PMC1121605

[B24] Metz J Hundertmark U Pevny I Vitamin C allergy of the delayed type Contact Dermatitis 1980 6 172 174 10.1111/j.1600-0536.1980.tb05592.x 7389324

[B25] Burns J Ouvrier RA Yiu EM Joseph PD Kornberg AJ Fahey MC Ryan MM Ascorbic acid for Charcot-Marie-Tooth disease type 1A in children: a randomised, double-blind, placebo-controlled, safety and efficacy trial Lancet Neurol 2009 8 537 544 10.1016/S1474-4422(09)70108-5 19427269

[B26] Toth C Poor tolerability of high dose ascorbic acid in a population of genetically confirmed adult Charcot-Marie-Tooth 1A patients Acta Neurol Scand 2009 120 134 8 10.1111/j.1600-0404.2008.01134.x 19154534

[B27] Huxley C Passage E Robertson AM Youl B Huston S Manson A Sabéran-Djoniedi D Figarella-Branger D Pellissier JF Thomas PK Fontés M Correlation between varying levels of PMP22 expression and the degree of demyelination and reduction in nerve conduction velocity in transgenic mice Hum Mol Genet 1998 7 449 458 10.1093/hmg/7.3.449 9467003

[B28] Ginter E Endogenous ascorbic acid synthesis and recommended dietary allowances for vitamin C Am J Clin Nutr 1981 34 1448 1451 7258136 10.1093/ajcn/34.7.1448

[B29] Reilly MM de JP Pareyson D 136th ENMC International Workshop: Charcot-Marie-Tooth disease type 1A (CMT1A) 8-10 April 2005, Naarden, The Netherlands Neuromuscul Disord 2006 16 396 402 10.1016/j.nmd.2006.03.008 16684603

